# On what basis are medical cost-effectiveness thresholds set? Clashing opinions and an absence of data: a systematic review

**DOI:** 10.1080/16549716.2018.1447828

**Published:** 2018-03-22

**Authors:** David Cameron, Jasper Ubels, Fredrik Norström

**Affiliations:** ^a^ Department of Public Health and Clinical Medicine, Umeå University, Umeå, Sweden

**Keywords:** WTP, willing-to-pay, international, systematic review, healthy adjusted life expectancy, HALE, C/E thresholds, cost-effective, decision-making, QALY

## Abstract

**Background:** The amount a government should be willing to invest in adopting new medical treatments has long been under debate. With many countries using formal cost-effectiveness (C/E) thresholds when examining potential new treatments and ever-growing medical costs, accurately setting the level of a C/E threshold can be essential for an efficient healthcare system.

**Objectives**: The aim of this systematic review is to describe the prominent approaches to setting a C/E threshold, compile available national-level C/E threshold data and willingness-to-pay (WTP) data, and to discern whether associations exist between these values, gross domestic product (GDP) and health-adjusted life expectancy (HALE). This review further examines current obstacles faced with the presently available data.

**Methods**: A systematic review was performed to collect articles which have studied national C/E thresholds and willingness-to-pay (WTP) per quality-adjusted life year (QALY) in the general population. Associations between GDP, HALE, WTP, and C/E thresholds were analyzed with correlations.

**Results**: Seventeen countries were identified from nine unique sources to have formal C/E thresholds within our inclusion criteria. Thirteen countries from nine sources were identified to have WTP per QALY data within our inclusion criteria. Two possible associations were identified: C/E thresholds with HALE (quadratic correlation of 0.63), and C/E thresholds with GDP per capita (polynomial correlation of 0.84). However, these results are based on few observations and therefore firm conclusions cannot be made.

**Conclusions**: Most national C/E thresholds identified in our review fall within the WHO’s recommended range of one-to-three times GDP per capita. However, the quality and quantity of data available regarding national average WTP per QALY, opportunity costs, and C/E thresholds is poor in comparison to the importance of adequate investment in healthcare. There exists an obvious risk that countries might either over- or underinvest in healthcare if they base their decision-making process on erroneous presumptions or non-evidence-based methodologies. The commonly referred to value of 100,000$ USD per QALY may potentially have some basis.

## Background

A prominent issue concerning many national healthcare systems today is how much to invest in new medical products, services, and intervention programs [1]. An integral part of this type of investment regards potential improvements in quality-adjusted life years (QALYs) and how much healthcare systems should be willing to spend for additional QALYs for their patients [1]. The cost-effectiveness (C/E) threshold, a tool used by countries to dictate the maximum financial investment a country or organization is willing to invest to give a patient an additional QALY, ranges greatly from country to country depending on methods and assumptions used [2]. Though not all countries use a formal C/E threshold system, the valuation of a QALY, when adjusted for inflation and purchasing power parity (PPP) to 2015 USD, can range from as little as 4419$ USD per QALY gained in Thailand to 173,971$ USD per QALY gained in Norway [3]. Though some argue that C/E thresholds are arbitrary and perhaps should be abandoned as a formal measure, two opposing approaches argue for the existence of formal, evidence-based C/E thresholds, though they differ in their reasoning and may produce two very different C/E threshold values [2,4]. Explicit cost-effectiveness thresholds act as a hard limit and are the major determinant in the decision-making process [5]. Conversely, implicit thresholds are not necessarily official ranges or values used by decision-makers, but can be inferred retrospectively by analyzing the cost-effectiveness of interventions previously evaluated by decision-makers [5–7]. With implicit thresholds, there exists greater potential for decision-makers to feel increased pressure to approve or reject certain drugs due to the zeitgeist of the current political landscape, regardless of the potential impacts on a population’s health which may cause social or political tension [8].

Those favouring an extra-welfarist approach argue in favour of using opportunity cost as a method of determining C/E thresholds [5,9,10]. According to this theory, the general public does not have the data or expertise to determine how resources can effectively be allocated to maximize the health of a population and the decision-making process should be reserved for experts. Given the nature of federal expenditures constrained within finite budgets, any direct investment in national healthcare is a draw from a different area; some indirect investments, to areas such as education or waste management, could result in larger increases to the average national level of health than investing directly into the healthcare system. Similarly, internal to healthcare systems, the funding of a new intervention imposes additional costs on restricted healthcare budgets and may require fund reallocation from other interventions and services elsewhere within healthcare systems [10,11].

Alternatively, C/E thresholds can be based on willingness-to-pay (WTP) per QALY values. This method is based on information about populations’ preferences so that they can be better reflected in the healthcare system. This welfarist approach argues that healthcare is financed through tax systems and thus a population’s preferences should be reflected onto how much value is placed on healthcare services [5,9,12]. Welfarists also argue that populations have the best available knowledge of how they value their own health and thus population preferences should be the basis of defining C/E thresholds (a discussion about WTP per QALY studies can be found in Appendix A).

Presently, there is no commonly agreed-upon value or method for determining C/E thresholds. Some federal health systems compare their own gross domestic product (GDP) per capita to the cost per QALY of new medical interventions when deciding to approve new drugs based on the World Health Organization’s (WHO) one-to-three times GDP per capita recommendation [13,14]. However, this recommended threshold is based on a long-standing misinterpretation and not on any methodological justification [3,15–20]. Also commonly referenced in American health economic literature is the value of 50,000$ USD per QALY. According to Grosse [21], this value stems from the cost of dialysis in the 1980s. Similarly, 100,000$ USD per QALY is often referenced as the suitable C/E threshold without justification. In US-based cost-utility analyses, 77.5% of all authors use either 50,000$ USD or 100,000$ USD per QALY as a reference point for cost-effectiveness [22].

According to the Grossman model of health economics [23], investment in healthcare systems faces decreasing marginal return to scale. In line with this model, countries with higher GDPs often have more funds available to invest in healthcare systems and may have a populace more interested in experiencing a higher level of health [23]. This results in rich countries being at particular risk of overinvesting in new, expensive medical interventions. Baker et al. [5] modeled the relationships between expenditure, opportunity cost, WTP, and C/E thresholds in healthcare investment, and illustrated diminishing returns to scale and the potential for an efficient C/E threshold where marginal cost (MC) equals marginal benefit (MB) (illustrated and further explained in Appendix C).

The aim of this systematic review is to describe prominent approaches to setting a C/E threshold, compile available national level C/E threshold and WTP data, and discern whether there are possible associations between C/E thresholds as well as WTP per QALY and other variables. This review will also discuss obstacles faced due to data limitations.

## Methods

**Figure 1.**
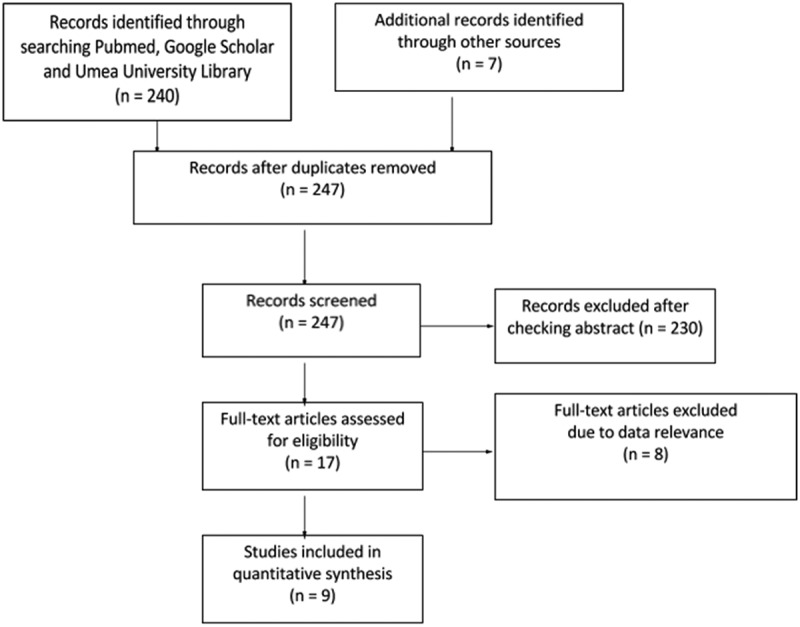
Stream diagram of C/E threshold article search.

A systematic search of the relevant literature, using Google Scholar, PubMed, and the Umeå University Library Catalogue, was conducted using the keywords ‘cost-effectiveness threshold’ and ‘decision making’ and ‘healthcare systems’ and ‘QALY’, published in English between 2010 and 2016 to exclude possible out-of-date data (Figure 1). The search was conducted by two reviewers working independently to create one database resulting in 240 papers. Four additional papers were identified after checking the references and ‘cited by’ sections of the papers identified in our searches. Based on recommendations by colleagues and to ensure that no grey literature was overlooked, the website domains of the Organisation for Economic Co-operation and Development (OECD), the Zorginsituut Nederland, and the Grupo de Ativistas em Tratamentos Portugal were searched using Google Domain search function for the term ‘cost-effectiveness thresholds’.

In total, 238 papers were screened due to our selection criteria. Studies that only justified the stated C/E value using WHO’s ‘recommendations’ were excluded while studies that stated their value as being ‘the most commonly accepted value for approving drugs in this country’ or cited government data were accepted. A further exclusion criteria pertained to some papers presenting values for countries as a whole, when in reality the country has different C/E thresholds for different provinces/states or for different areas of medicine [3]; these were filtered on a case-by-case basis (explained in Appendix A). From the resulting papers, 17 countries were identified from 9 unique sources to have formal C/E thresholds within our inclusion criteria. The C/E thresholds of England, Thailand, and Ireland are explicit, while the others are implicit.

**Figure 2.**
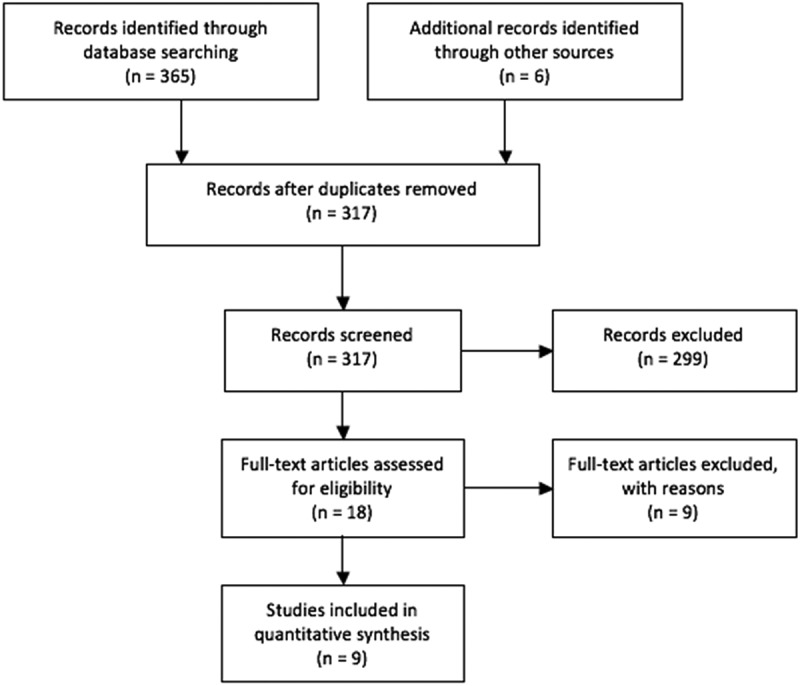
Stream diagram WTP per QALY studies.

A separate search was conducted through Pubmed, Google Scholar, and the Umeå University Library Catalogue for the keywords ‘ “Willingness to pay per QALY” country’. The search was conducted by two reviewers working independently to create one database. Only results published in English between the years 2000 and 2016 were included (Figure 2).

WTP per QALY studies were filtered using several criteria. It was required that the participants in each study be representative of the general population, the sample size be greater than 100 [24], and the diseases on which the health states are based on should be unknown to the participants (as further described in Appendix A) [25]. From our initial search, six articles were identified as relevant to our research question. An additional six articles were identified through the references of articles found in our initial search. Of these six additional articles, three were determined to be duplicates. This resulted in a total of 9 studies meeting our criteria, comprising WTP per QALY data for 13 countries.

Data are presented in 2015 US dollars PPP. Historical currency exchange rates, inflation rates, GDP per capita, and Purchasing Power Parity (PPP) were calculated for all retrieved data with the XE Online Historical Currency Converter [26], the US Inflation Calculator [27], and the World Bank Online Database for GDP per capita [28] and PPP conversion [28,29], respectively. Information regarding Taiwan’s PPP and inflation was taken from the CIA world factbook [30], as data were unavailable through the World Bank website.

Though many explicit thresholds are not frequently updated or adjusted [24], capturing the financial value of the threshold at the time data were published best reflects the values used in the decision-making process that determined the C/E threshold. Health-adjusted life expectancy (HALE) was sourced from the Global Burden of Disease study (2010) [25] and was selected as a measure of national average health due to its ability to compare health between countries and its extensive use in health economic literature. Potential linear correlations of WTP per QALY with HALEs, GDP per capita, and C/E thresholds were investigated (figures in Appendix B). Non-linear (exponential and polynomial) correlations of C/E thresholds with HALEs and GDP per capita were investigated because non-linear relationships were expected [5,31]. All the correlations were calculated with Microsoft® Excel® 2016 (Microsoft, Redmond, Washington, United States).

## Results

C/E thresholds are presented in Table 1; extra notes have been added to point out particular aspects of certain studies. Some countries without publicly available C/E thresholds have published official justifications for not using these measures, while most simply have no publicly available data [32,33].

**Table 1.** The results of the review of the available C/E thresholds by country.

Country	Cost-Effective Threshold (2015 USD PPP)	Notes	Health-Adjusted Life Expectancy (HALEs)	GDP per capita (2015 USD)	Study
Australia	63,096	Not a clear threshold, 51% of interventions rejected at this ICER or lower	70.10	46,223	Paris, Belloni (2013)
Belgium	180,653	Implicit	68.55	42,578	Paris, Belloni (2013)
Brazil	27,620	Implicit, per life years	63.85	15,838	Schwarzer et al. (2015)
Canada	98,183		69.60	44,057	Paris, Belloni (2013), Jaswal (2013)
Czech Republic	29,015	3x GDP/capita	67.20	30,407	Kowalczuk et al. (2015), Gulacsi et al. (2014)
Hungary	25,473	Implicit, 3x GDP	64.20	24,721	Gulacsi et al. (2014)
Ireland	84,094	Explicit	68.85	48,755	NCPE (2009–2016)
Japan	83,938	‘Frequently referred to’	73.05	36,426	Shiroiwa et al. (2013)
South Korea	23,124	Implicit and societal perspective, GDP per capita used as reference value	70.25	34,356	Paris, Belloni (2013)
Netherlands	132,340	Some orphan drugs are exception	69.05	47,663	Zorginsituut Nederland
Norway	173,971	Implicit. ‘[S]evere illnesses and orphan medicines are not supposed to be treated differently.’ Even though Norway does not have a clear C/E, this WHO-inspired value may be representative of Norway’s C/E	68.00	64,856	Paris, Belloni (2013)
Poland	19,006	3x GDP/capita. ‘There is no clear relationship between C/E of drug and whether it is improved for reimbursement.’ Many drugs are rejected for other reasons.	66.05	24,745	Kowalczuk et al. (2015)
Portugal	31,890	‘Anecdotal evidence suggests that the Portuguese National Authority of Medicines (Infarmed) adopts an informal threshold of 30,000/QALY.’	68.55	28,393	Yazdanpanah et al. (2013)
Sweden	50,173	Uses societal perspective	69.60	45,183	Paris, Belloni (2013)
Thailand	4419	Explicit	65.25	15,735	Schwarzer et al. (2015)
UK	65,871	Explicit	68.60	39,762	Paris, Belloni (2013)
USA	100,000.00	This value is often referred to as both QALYs gained and DALYs averted	67.85	54,630	Neuman (2014)

Data [3,13,22,34–37,51].

**Figure 3.**
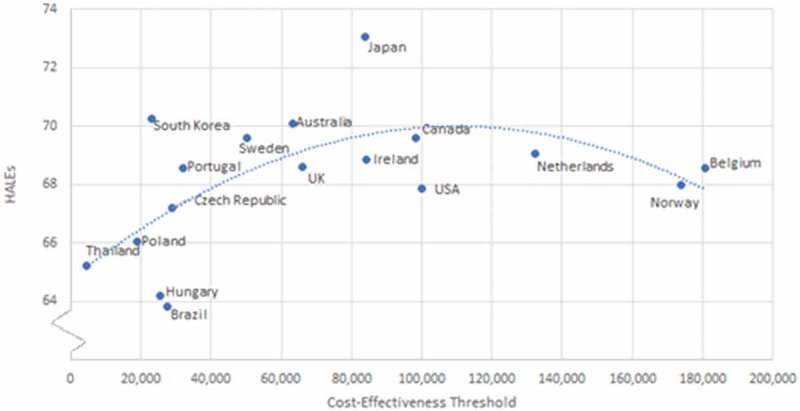
Cost-effectiveness thresholds in purchasing power parity adjusted 2015 US dollars compared to healthy adjusted life expectancy by country.

The PPP-adjusted C/E thresholds (Table 1), are correlated with HALEs; a quadratic relationship may be seen with a polynomial correlation of 0.633 (R = 0.63) with an apex of approximately 100,000$ USD per QALY (Figure 3).

**Figure 4.**
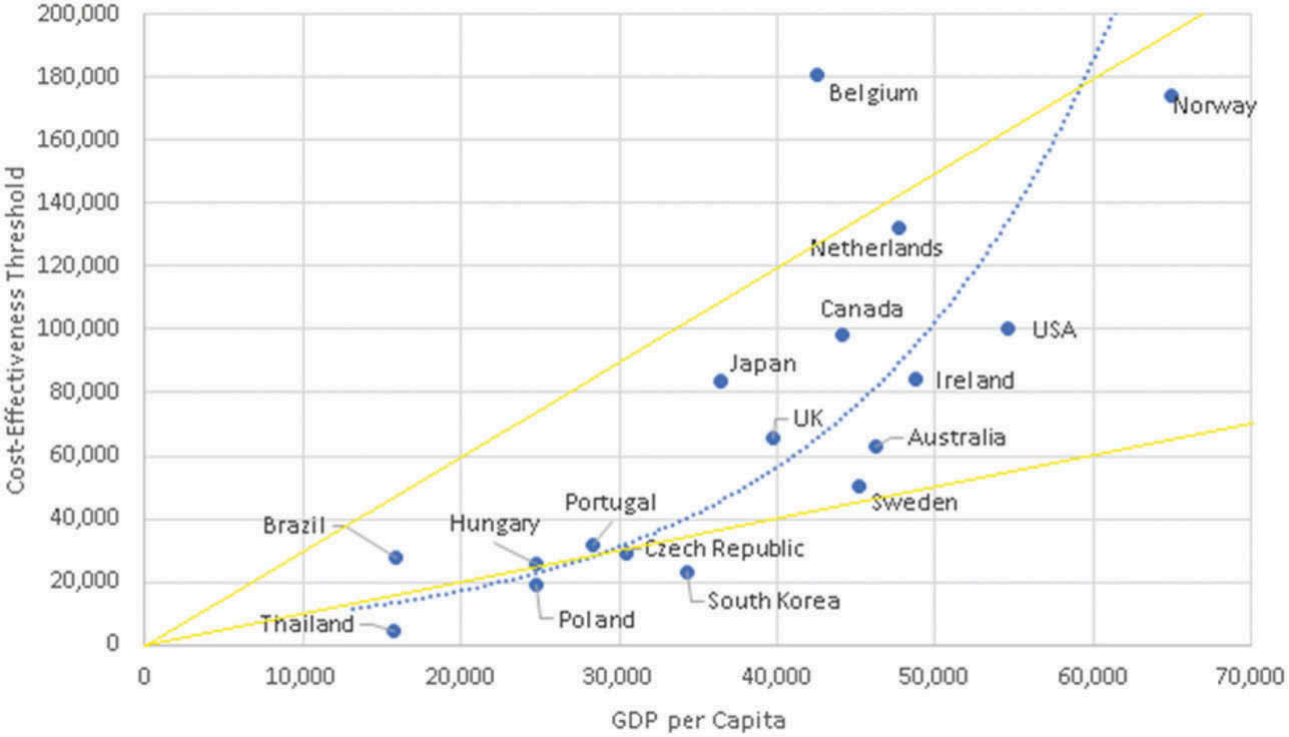
Cost-effectiveness threshold plotted against GDP per capita.

A relationship can also be seen between the C/E thresholds and GDP per capita (Figure 4). Most countries with formal C/E thresholds fall within WHO’s ‘recommendation’ of one-to-three times GDP per capita and are from OECD countries. Two additional solid lines have been added to this graph to illustrate which countries have thresholds that fall within WHO’s ‘recommendation’ of one-to-three times GDP. The dotted line expresses a line of best fit with a polynomial correlation (R = 0.84).

WTP data are amalgamated in Table 2. The average WTP per QALY was found to be $77,509, with a range from 1415$ USD to 123,695$ USD, and a standard error of $15,193. No correlations could be identified between the WTP per QALY of a country and C/E thresholds, GDP per capita, and HALEs. Given the low comparability between WTP studies due to differing methodology, all other results pertaining to WTP are displayed in Appendix B.

**Table 2.** WTP per QALY review table.

Country	Study Type: WTP	Study Type: QALY	Date of data	Results^a^	Mean Results (2015 USD PPP)	National average results for country if multiple studies (2015 USD PPP)	Study
Australia	Ex post With starting point bias	Scale-like per extra QALY (disease is described, and then how much would you pay to get rid of disease)	2008	64,000$ AUD	67,918	N/A	Shiroiwa et al. (2010)
China	Ex ante	Double-bounded dichotomous choice	2009	4700–7400$ USD	6641	N/A	Zhao et al. (2010)
Denmark	Ex ante and ex post	Time trade-off, Standard gamble	2007	32,754–87,752€ EUR	91,974	30,658	EuroVaQ
Denmark	Ex post	Time trade-off	2012	3040–107 688€ EUR	75,264	30,658	Gyrd-hansen, Kjær (2012)
Denmark	Ex post (not to 1 full QALY)	EQ5D (EuroQol) health states dichotomous choice	2003	88,000kr DKK	15,782	30,658	Gyrd-Hansen (2003)
Hungary	Ex post and ex ante	Time trade-off, Standard gamble	2007	51,145–112,234€ EUR	124,695	N/A	EuroVaq
Japan	Ex post With starting point bias	Scale-like per extra QALY	2008	5,000,000¥ JPY	51,982	51,655	Shiroiwa et al. (2010)
Japan	Ex post	Double-bounded dichotomous choice	2013	20,000–80,000$ USD	51,327	51,655	Shiroiwa (2013)
Korea (Republic of Korea)	Ex post, with starting point bias	Scale-like per extra QALY	2008	68,000,000₩ KRW	75,209	N/A	Shiroiwa et al. (2010)
Netherlands	Ex ante and ex post	Time trade-off, Standard gamble	2007	55,274–180,295€ EUR	179,793	N/A	EuroVaq
Spain	Ex ante and ex post	Time trade-off, Standard gamble	2007	92,488–178,527€ EUR	206,846	103,423	EuroVaq
Spain	Ex post and ex ante	Standard gamble, using EQ5D health states	2009	27,192€ EUR	42,035	103,423	Pinto-Prades et al. (2009)
Sweden	Ex-post and ex ante	Time trade-off, Standard gamble	2007	50,712–168,152€ EUR	167,04	N/A	EuroVaq
Taiwan	Ex post With starting point bias	Scale-like per extra QALY	2008	2,100,000$ NT$	76,414	N/A	Shiroiwa et al. (2010)
Thailand	Ex ante Corrected for starting point bias	Time trade-off and visual analog scale	2013	26,000–137,000฿ baht	918	1415	Thavorncharoensap et al. (2013)
Thailand	Ex post Corrected for starting point bias	Time trade-off and visual analog scale	2013	59,000–285,000฿Baht	1912	1415	Thavorncharoensap et al. (2013)
UK	Ex post With starting point bias	Scale-like per extra QALY	2008	23,000£ GBP	50,683	74,321	Shiroiwa et al. (2010)
UK	Ex ante and ex post	Time trade-off, Standard gamble	2007	50,524–77,824£ GBP	97,959	74,321	EuroVaq
USA	Ex post With starting point bias	Scale-like per extra QALY	2008	$62,000$ USD	68,573	48,429	Shiroiwa et al. (2010)
USA	Ex post	Time trade-off, visual analog scale, and WTP	2003	12,500–32,200$ USD	28,285	48,429	King et al. (2005)

^a^If results were presented as a range by the authors, the mean of the range was taken.

Data [35,38–41,50,51,55–57].

## Discussion

Given that only 17 countries have had data published regarding their C/E threshold, it seems that formal and methodological C/E thresholds are a neglected and non-transparent part of decision-making in many countries. Decision-makers may be averse to basing politically sensitive decisions on a single summary measure alone; issues regarding the validity of cost-effective ratios and QALYs may encourage decision-makers to rely more on their own judgment [42]. Many countries lack a formal explicit threshold and use alternative strategies that result in an implicit C/E threshold. Some countries have specified justifications for abstaining from their use while most do not seem to have any reasoning at all.

Germany is an example of a country that does not use thresholds [17]. Federal policymakers in Germany assert that C/E thresholds are not compatible with German law and history; however, in recent years decision-makers are slowly introducing some economic evaluation into their decision-making process [17]. Other countries do not give an explanation at all. This may be due to a low priority being given to setting a C/E threshold, difficulties in identifying and presentation C/E thresholds, or a lack of health economic expertise.

The apex of the curve correlating HALEs and C/E thresholds may be illustrating the opportunity cost and diminishing marginal returns in healthcare investment as discussed by Baker et al. [5]. Coincidentally, the approximate apex of the curve (100,000$ USD per QALY) is the same commonly cited value that is believed to be based on no actual evidence, suggesting that it may potentially be a reasonable reference point for C/E thresholds. Though limited by the number of observations, this figure also illustrates the potential for overly inflated C/E thresholds. Some countries have relatively high C/E thresholds but are experiencing overall lower HALEs. Tertiary variables (GDP per capita, total healthcare expenditure, behavioural factors, etc.) could potentially be confounding these results. As more countries refine and formalize their methods of approving new medical interventions, and more data become available, possible correlations with C/E thresholds may be further examined. Almost all identified C/E national thresholds fall within the WHO guidelines of one-to-three times GDP per capita. Although these guidelines have been shown to be largely arbitrary, they still may be influencing decision-makers in C/E threshold setting or coincidently reflect the results of independent C/E setting processes.

From the welfarist perspective, the average for WTP per QALY of 77,509$ USD lays between the commonly used C/E thresholds of 50,000$ USD – 100,000$ USD per QALY and is similar to the apex of the curve seen in Figure 3. In theory, a strong argument can be made to base C/E thresholds on population preferences; but, in practice, there are too many methodological problems with WTP per QALY studies to make any meaningful decisions based on the presently available data [21,43]. An opportunity cost based approach is an ideologically promising way of setting a C/E threshold; however, data regarding opportunity cost and calculating the impact of specific programs may be unduly complex [5].

In the case of decision-makers subscribing solely to a rigid C/E threshold, ethical issues and inequalities may arise [44]. For example, a patient with a base QALY of 0.2 who is expected to improve to 0.4 through an intervention would be evaluated the same as someone with a base QALY of 0.8 who is expected to improve to 1.0. Although these QALY gains are numerically ‘equal’, Bobinac et al. [44] argue that this exemplifies an inequity since one of these patients will value the QALY gain far more than the other. This example suggests that determining C/E thresholds from a purely economical perspective may lead to unwanted inequities and other negative results. While setting C/E thresholds solely based on economic arguments potentially may prevent some inefficiencies, there are clearly other benefits from considering other factors in the decision-making process. C/E thresholds can aid decision-makers when appraising evidence, without being the sole metric. Sweden, for example, also considers ‘the human value principle’ (those with the most pressing medical needs should be prioritized) and ‘the need and solidarity principle’ (respecting the equal value of all human life) in their decision-making process [45].

Practical examples of conflicting opinions between the public and decision-makers can be found in England and the Netherlands. In England, conflicting approaches regarding the funding of new treatments and the valuation of health have led to high-profile clashes of opinion. A recent example of the conflict between federal C/E thresholds and public opinion took place in England where the National Institute for Health and Clinical Excellence (NICE) recommended the rejection of funding for five drugs because their cost far exceeded a C/E threshold [46]. Consequently, the National Health Service (NHS) was not obliged to compensate for these drugs. This decision resulted in patients suffering from chronic lymphocytic leukaemia and mantle cell lymphoma not having access to medication that could improve their quality of life [47].

In the Netherlands, the College voor Zorgverzekeraars, a Dutch institution similar to NICE, recommended ending compensation for the medicines used to treat Pompe disease and Fabry disease [8]. Both diseases are treatable, though the medication is relatively expensive, costing up to €700,000 per patient per year. After ensuing societal uproar, the Dutch Minister of Health, Welfare, and Sports decided to renew compensation for these medications [8]. The national turmoil that resulted in the Netherland’s initial decision illustrates how a population’s WTP per QALY may not align with current C/E thresholds. Given the high price of this drug, this example demonstrates the mismatch between opportunity cost in healthcare investment and a population’s WTP per QALY.

A limitation of this study is the lack of available data and information on C/E thresholds available. This presents difficulties in conducting in-depth analyses on how C/E thresholds influence the average health of a nation. After a systematic review of the literature, as well as incidental personal communication with healthcare experts of countries, we found that most countries do not have publicly available data regarding their drug-approval decision-making process.

Our data review did not reveal how many countries refrain from the use of formal methodologies for approving new medical interventions and data for some countries may have been missed. Since the present research has a limited number of data points, the inclusion of new, additional data could influence our results. Further research is needed to study the possible associations potential relationships described in our study.

Few WTP per QALY studies have been conducted at the national level (appropriate data for only 13 countries were found) and many took differing approaches. Due to conflicting methods in data collection and analysis the comparability of the results of these studies is limited. If a large-scale, standardized, international WTP per QALY study is conducted, researchers would be better equipped to analyze whether relationships exist between national average individual valuation of health, national average level of health, national C/E threshold guidelines, and other factors.

## Conclusion

Decision-makers need relevant data and strategies in order to make evidence-based decisions when setting C/E thresholds; however, the quality and quantity of data available regarding national average WTP per QALY, opportunity costs, and C/E thresholds is poor in comparison to the importance of adequate investment in healthcare. Given that large decisions regarding investment in new medical interventions are made without appropriate economic justification, the need for further research and data collection is clearly evident. A potential exponential relationship was observed between C/E thresholds and GDP per person, and a potential quadratic relationship was observed between HALEs and C/E thresholds; these relationships may warrant further study if more data become available. By further studying these relationships, researchers may create useful strategies for determining C/E thresholds and whether WHO’s ‘recommendation’ of one-to-three times GDP or the commonly cited value of 100,000$ USD per QALY have merit.

## Figures and Tables

**Figure B1.**
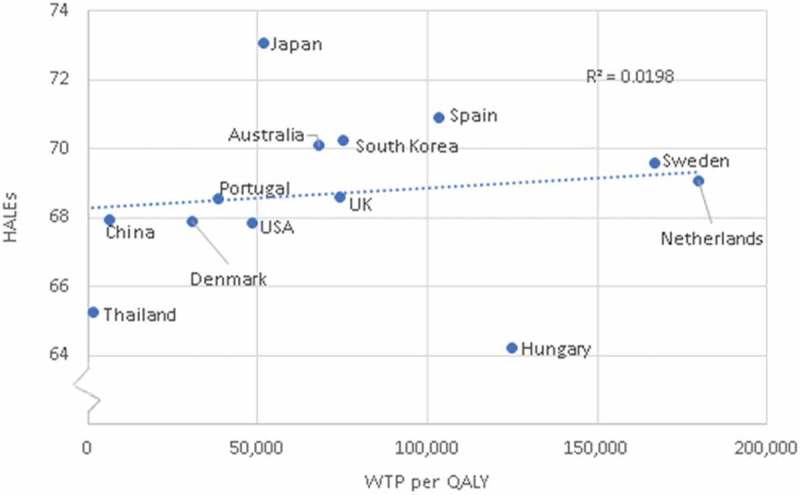
WTP per QALY plotted against HALEs by country. The Y-axis has been truncated for clarity.

**Figure B2.**
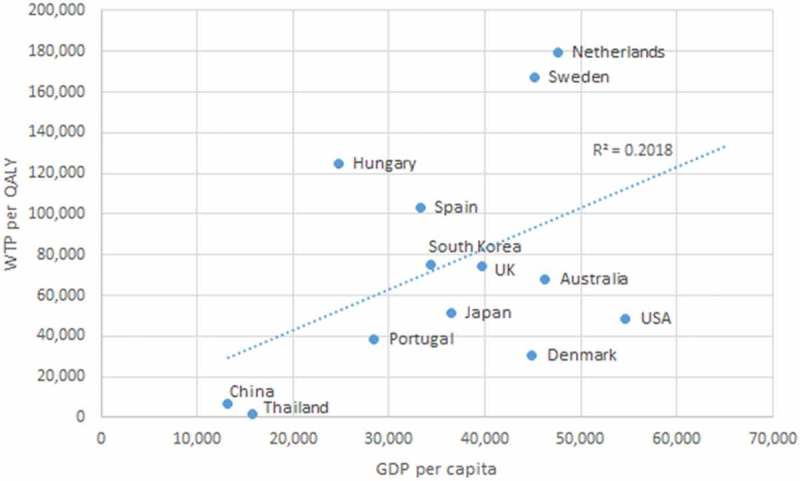
GDP per capita plotted against WTP per QALY by country.

**Figure B3.**
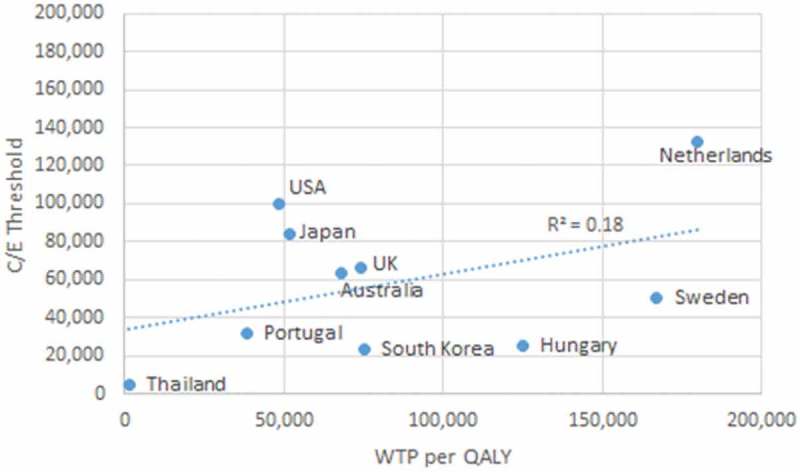
C/E Threshold plotted against WTP per QALY by country.

**Figure C1.**
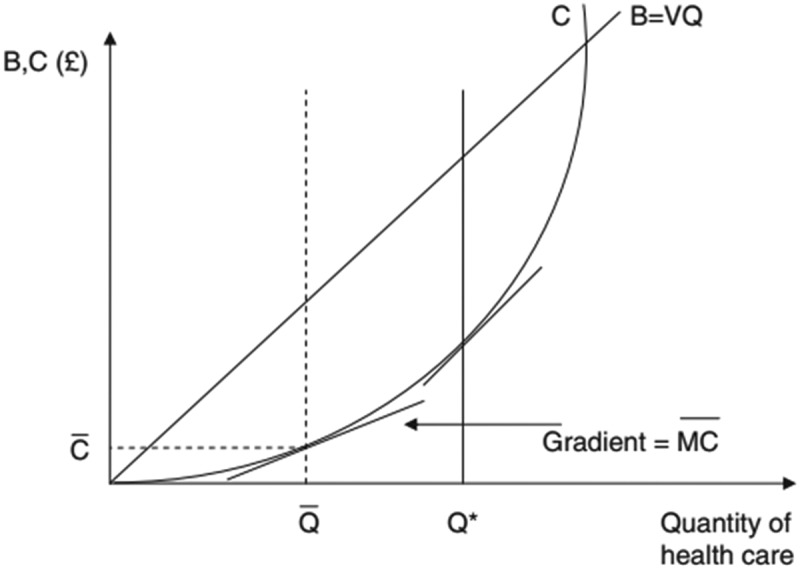
Expenditure and opportunity costs in healthcare investments (adapted from Baker et al. [5], used with permission from authors and publisher).

## Paper context

Cost-effectiveness analysis guide decision-makers in resource allocation decisions. There exist different philosophical approaches and justifications in setting a threshold. Given the finite and often strained budget of health systems, accurately setting cost-effectiveness thresholds can have a large impact on a nation’s average health. This review gives an overview of the different theories and justifications used for setting cost-effectiveness thresholds and an analyses of the existing data to find possible correlations between health, cost-effectiveness thresholds, national willingness-to-pay per quality-adjusted life year and GDP per capita.

## Appendix A

### Basis of using WTP per QALY

According to the *Blackwell Guide to Medical Ethics* [48], many theorists argue that governments and those who use public funds have a responsibility to act in accordance with the preferences of their population. Given the lack of an objective tool or economic measure for setting a C/E threshold, public opinion should certainly be considered in the decision-making process. WTP per QALY surveys and studies allow researchers to better understand how a population values health and may aid researchers in making more informed decisions that better reflect public opinion.

The current methodologies for interpreting individual valuations of a QALY are riddled with flaws. In [21] it is stated that ‘there is mounting evidence that the average individual WTP for QALYs resulting from improvements in health status from relatively minor conditions is lower than the WTP gains from lifesaving interventions’. In current methodologies, it seems individuals don not always treat every QALY equally. These flaws may further compound issues created by contextual and environmental variables that confound WTP studies.

### Instrumental approaches in the valuation of a QALY

The amount a disease impacts an individual’s utility, or QALY weight, is elicited using four principal methodologies: the EuroQol questionnaire (EQ-5D), visual analog scales, time trade-off, and standard gamble. These methods all serve the purpose of collecting information about the utility value of different health states of individual participants.

The first method of measuring the utility of a health state is by using the EQ-5D questionnaire [49]. This questionnaire contains questions on a broad spectrum of health states which all have their own specific QALY weight. These standardized health states can be used in various different ways for WTP per QALY research. Usually, several specific health states are chosen by researchers and presented to participants. Then, a participant is asked to choose how much he or she is willing to pay to move from these states to reach a better health state; this elicits a WTP per QALY value.

Secondly, in the visual analog scale method, a participant is asked to look at a scale with a range (0–10, 0–100 etc.), where the upper number is defined as perfect health and the lowest number as death. Then, the participant is asked to rate hypothetical health states chosen by the researchers on this scale. The value that the participant selects on the time scale determines the QALY valuation [50].

Thirdly, the standard gamble method presents patients with both a hypothetical health state and a cure to achieve perfect health. However, the cure also has a certain chance to kill the participant when taken. The participant then has to decide whether or not to risk death in order to become cured. Different health states and different cures with different risks of death are then used to determine the QALY of that specific health state.

Fourthly, the time trade-off method participants are presented with a hypothetical disease or health state which they need to live with for a certain amount of time [50]. Then, participants are asked how many years of their life they would exchange in order to suddenly improve to their previous, higher health state. The result of this question is then divided by the amount of time the researcher proposed in its hypothetical health state, which results in a ratio which is used to determine the QALY value.

In order to measure the monetary value of a QALY, two main methods of eliciting the WTP per QALY can be used: the double-bounded dichotomous choice method and the bidding strategy the ex-post or bidding game method [50]. In the bidding game method, a participantis asked to imagine being in a lower health state (a health state with a lower QALY score) for acertain amount of time without treatment and then return tonormal health. Then, the participant is informed of a treatment that would immediately treat the negative health state. Participants are then asked how much they would hypothetically pay out of their own pocket for that respective treatment. In the double-bounded dichotomous choice, respondents are asked if they would say yes for certain bid values to get a certain treatment that will bring them back to health. Depending on the answer, after a yes a higher bid value would be shown and after a no a lower bid value until the price that the respondent is willing to pay is elicited [51].

These answers are not necessarily systematically varied, because the answers produced by all these methods are influenced by the same external factors. The first factor to note is the difference in income levels: people with higher incomes are willing to pay generally more for a good, in this case a QALY. The second factor is life expectancy: a person with a high life expectancy values the gain of one QALY less than a person with low life expectancy, since one QALY is worth relatively more for a person with low life expectancy compared to a person with high life expectancy [52]. The third key factor is how much an individual values a life year. There are differences in how much value people place on their health and life compared to other goods. An individual might, for example, place great value on smoking for several reasons even though it has a negative effect on health, while another person does not [53].

### Methodological distinctions in WTP per QALY studies

Schwarzer et al. [4] found a relationship between a population’s average ability-to-pay (ATP) per QALY and their WTP per QALY. Furthermore, it has been found that poverty influences an individual’s WTP for adequate health care [54]. Researchers studying WTP per QALY observed vastly different results when using different methodologies and population groups [55–57]. Six distinctions were identified that explain this variability and unreliability in outcomes in the WTP per QALY studies.

The first distinction is between studies that focus their questions around WTP for health gains, also called the ex-post approach [58], and studies that focus their questions on the WTP for preventing a potential health loss, also called the ex-ante approach [58]. The ex-post or bidding game is the most common method used in WTP per QALY studies [50]. The bidding method works as follows: a participant of the study is asked to imagine experiencing a lower health state (a health state with a lower QALY score than one) for a certain amount of time without a treatment (after that amount of time they will return to normal health). Then, the participant is informed of a treatment that would immediately treat the negative health state so that the participant does not have to wait a specific amount of time to return to normal health. Participants are then asked how much they would pay out of their own pocket for that respective treatment. In contrast, in the ex-ante or risk variant approach participants are asked how much they value staying in a certain health state given a particular chance that they lose it. For example, a participant is presented with a hypothetical situation where he or she is fully healthy (a QALY of one), but has a 50% chance of loosing that health state and moving to a lower QALY state of 0.8 for a certain amount of time. The participant is then asked how much value he or she places on removing this risk. The outcomes of these studies may differ, as ex-post studies do not take into account a risk factor and people have full information about their health state. It can thus be argued that the ex-ante method of valuing WTP per QALY gives more realistic answers, as in real life certainty about health states does not exist; one never knows when one will get sick and what kind of diseases will be contracted.

The second distinction is between studies that focus on the increase and decrease of quality of life and studies that focus on the extending or shortening of life [12]. While in theory this should not make a difference in calculations with QALYs (a two-year life extension of 0.5 QALY has theoretically the same worth as a one-year life extension of 1 QALY), there is a difference found in practice. Studies show that the WTP for extending or preventing a shortening of life is greater than an increase or prevention of decrease in quality of life [12]. This means that while in theory every QALY has the same value, in practice it seems that the general population values some QALYs more than others.

A third distinction can be made between studies that study large QALY differences and small QALY differences [12]. Larger QALY differences result in relatively lower WTP for that QALY. This means that the relation between WTP per QALYs and difference in QALYs is not proportional. A possible explanation for this problem is that the general population has trouble internalizing, conceptualizing, and valuing QALYs. This begs the question of whether WTP per QALY studies are representing the actual values the general population places on health; perhaps the valuation of QALYs should be expressed and measured in non-linear terms.

A fourth distinction can be made between studies that focus on sampling from patient populations and general populations. When patient populations are asked about an increase in QALYs, their WTP is on average lower than the general population’s. A possible reason for this is the fact that the expected utility of a disease by the general population is lower than the actual utility of having that disease [59,60].

A fifth distinction between WTP per QALY studies can be made between studies that take a individual or a societal focus. Studies with an individual focus study how much an individual is WTP for his or her own health while studies with a societal focus either take into account altruistic motives: how much does a person value the health of a family member or a stranger? Most studies are conducted with the individual focus; however, in recent years the interest in societal WTP per QALY is rising [61]. The added value of approaching the WTP per QALY from a societal perspective is that most healthcare systems in most European countries are based on the philosophies of solidarity and on sharing the healthcare costs as a collective [62]. This means that studying WTP per QALY from a societal perspective gives answers that are more applicable to the philosophical principles of most healthcare systems [61].

## Appendix B

Relationships between WTP per QALY with HALEs (Figure B1), GDP per capita (Figure B2), C/E thresholds (Figure B3) were investigated; no strong relationships were found.

## Appendix C

In Figure C1, Baker et al. [5] visualized expenditure and opportunity cost in healthcare investment. Q, quantity of healthcare provided, is plotted on the horizontal axis and the corresponding total cost of this healthcare, C, as well as its total benefit, B, are plotted on the vertical axis. A diminishing return in health benefits can be seen as the quantity of healthcare is increased when Q > Q*. The total benefit Benefit, B, is a function of both the quantity of healthcare provided as well as V, the value a society gives to this healthcare (i.e. willingness-to-pay [WTP] per QALY). Thus, ideal healthcare expenditure occurs where marginal cost is equal to marginal benefit and C/E thresholds should be set that allows this to occur. According to this graph, ideal healthcare expenditure will be at Q*, where marginal cost (MC) = marginal benefit (MB). Given that a higher threshold leads to higher healthcare expenditure, approving or rejecting new treatments that greatly change Q may lead to inefficiencies in resource allocation and waste [5]. According to this model, thresholds should be set where the marginal benefit gained by a new treatment is equal to or greater than the marginal cost of implementing it. This figure illustrates both the welfarist and extra-welfarist perspective by defining benefit (B) along the y-axis in terms ofhealthcare provision as well as a function of WTP. For extra-welfarists, this graph demonstrates that by considering opportunity costs when determining C/E thresholds, an economically efficient outcome is possible. Likewise, if *V*, per unit of health care is a multiple (k > 1), then an efficient allocation of healthcare could be set where marginal benefit/cost ratio of a medical intervention is equal or greater than k [5].
